# Supplementary material for: NGFI-A Binding Protein 2 Promotes EGF-Dependent HNSCC Cell Invasion
Source: Cancers (Basel). 2019 Mar 6;11(3):315. doi: 10.3390/cancers11030315 (PMC6468740; doi:10.3390/cancers11030315)
Supplement: Supplementary file 1 [file cancers-11-00315-s001.pdf]

cancers

Supplementary Materials: NGFI-A Binding Protein 2 Promotes EGF-Dependent HNSCC Cell Invasion

Jinkyung Kim, Sung-Min Kang, Su Young Oh, Heon-Jin Lee, Inhan Lee, Jae Chan Hwang and Su-Hyung Hong

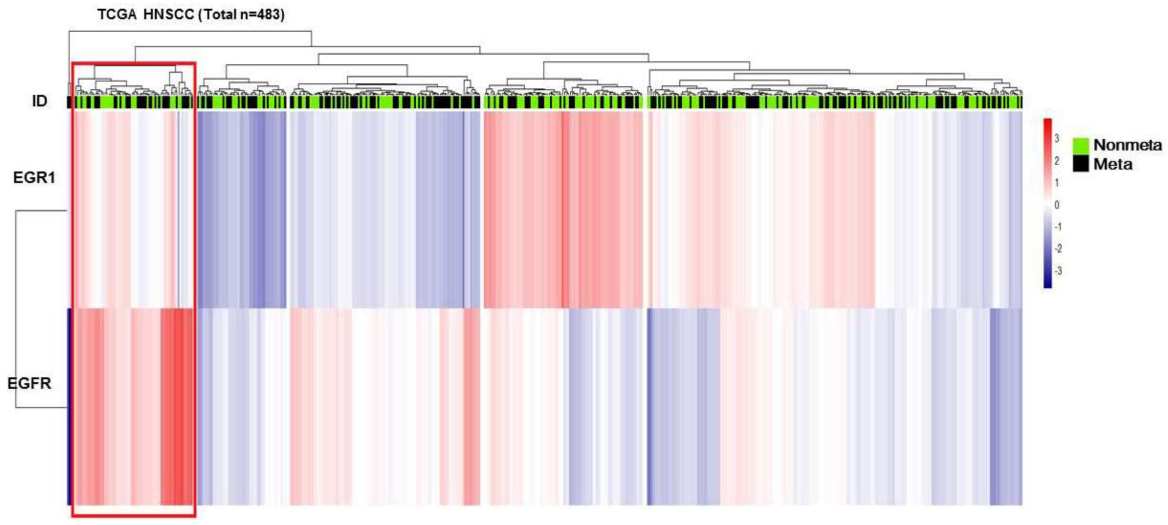

Figure S1. Heatmap representation of the relative mRNA expression levels of *EGFR*/*EGR1* in the TCGA population of head and neck squamous cell carcinoma (HNSCC) specimens. Unsupervised hierarchical clustering with distance measured based on Pearson correlations was performed on log_2-transformed FPKM-UQ normalized *EGFR*/*EGR1* mRNA expression data for all patients. Metastatic and non-metastatic patient tumor samples were annotated with green and black bars, respectively.

© 2019 by the authors. Licensee MDPI, Basel, Switzerland. This article is an open access article distributed under the terms and conditions of the Creative Commons Attribution (CC BY) license (http://creativecommons.org/licenses/by/4.0/).
